# Chemotherapy response prediction with diffuser elapser network

**DOI:** 10.1038/s41598-022-05460-z

**Published:** 2022-01-31

**Authors:** Batuhan Koyuncu, Ahmet Melek, Defne Yilmaz, Mert Tuzer, Mehmet Burcin Unlu

**Affiliations:** 1 Department of Computer Engineering, Bogazici University, Istanbul, 34342 Turkey; 2 Department of Management, Bogazici University, Istanbul, 34342 Turkey; 3 Department of Physics, Bogazici University, Istanbul, 34342 Turkey; 4 Center for Life Sciences and Technologies, Bogazici University, Istanbul, 34342 Turkey; 5 Hokkaido University, Global Station for Quantum Medical Science and Engineering, Global Institution for Collaborative Research and Education (GI-CoRE), Sapporo, 060-8648 Japan

**Keywords:** Computational models, Mathematics and computing

## Abstract

In solid tumors, elevated fluid pressure and inadequate blood perfusion resulting from unbalanced angiogenesis are the prominent reasons for the ineffective drug delivery inside tumors. To normalize the heterogeneous and tortuous tumor vessel structure, antiangiogenic treatment is an effective approach. Additionally, the combined therapy of antiangiogenic agents and chemotherapy drugs has shown promising effects on enhanced drug delivery. However, the need to find the appropriate scheduling and dosages of the combination therapy is one of the main problems in anticancer therapy. Our study aims to generate a realistic response to the treatment schedule, making it possible for future works to use these patient-specific responses to decide on the optimal starting time and dosages of cytotoxic drug treatment. Our dataset is based on our previous in-silico model with a framework for the tumor microenvironment, consisting of a tumor layer, vasculature network, interstitial fluid pressure, and drug diffusion maps. In this regard, the chemotherapy response prediction problem is discussed in the study, putting forth a proof of concept for deep learning models to capture the tumor growth and drug response behaviors simultaneously. The proposed model utilizes multiple convolutional neural network submodels to predict future tumor microenvironment maps considering the effects of ongoing treatment. Since the model has the task of predicting future tumor microenvironment maps, we use two image quality evaluation metrics, which are structural similarity and peak signal-to-noise ratio, to evaluate model performance. We track tumor cell density values of ground truth and predicted tumor microenvironments. The model predicts tumor microenvironment maps seven days ahead with the average structural similarity score of 0.973 and the average peak signal ratio of 35.41 in the test set. It also predicts tumor cell density at the end day of 7 with the mean absolute percentage error of $2.292\pm 1.820$.

## Introduction

Tumors need their blood supplies to grow beyond the size of 1–2 $\hbox {mm}^3$ in diameter and meet the needs of oxygen and other nutrients. For this reason, tumors stimulate angiogenesis, a process in which tumors form the new blood vessels from pre-existing ones by secreting the various growth factors and, most importantly vascular endothelial growth factor (VEGF). The angiogenic switch between proangiogenic and antiangiogenic factors is activated for tumor progression and metastases^1^. Due to tumor-induced angiogenesis, the newly developed vessels have a leaky and disorganized structure accompanying a microenvironment identified by hypoxia, acidosis, and increased fluid pressure^2^. As a result, this structurally and functionally abnormal tumor vascular network leads to heterogeneous and inadequate drug distribution inside tumors.

The chaotic architecture, high vascular permeability of tumor vessels, and lack of functional lymphatics lead to elevated interstitial fluid pressure (IFP), creating a barrier for the transport of therapeutic agents and nanoparticles^3,4^. IFP is uniform across the tumor almost equal to microvascular pressure (MVP), but it drops precipitously at the tumor boundary. Therefore, the absence of a pressure gradient along the vessels hampers the penetration of cytotoxic drugs to the interior parts of tumors transported by convection^3^.

Antiangiogenic agents are mainly utilized to prevent tumor growth in size or metastases to another organ by depriving the tumor of the blood supplies it needs. Normalization of tumor vasculature by using antiangiogenic agents is a widely used treatment modality in cancer therapy. Antiangiogenic agents maintain the balance between proangiogenic and antiangiogenic factors, similar to healthy tissues. Furthermore, these agents normalize the structure and function of the tumor vascular network transiently by inducing reduced vessel diameter and decreased vessel wall permeability. This process triggers tumor vessels to be more useful for the delivery of drugs as well as oxygen and nutrients to the targeted cancer cells^2,5^. Vascular normalization improves convective transport of drug particles with a decrease in IFP, thereby inducing pressure gradients across vessel walls^1,6^.

Antiangiogenic agents can act directly on the tumor vasculature^7^; also, several preclinical and clinical studies show that the application of antiangiogenic agents together with chemotherapy drugs provides beneficial results with increased therapeutic outcomes^8–10^. The combination therapy can enhance the delivery of therapeutic agents to the interior parts of tumors^6^. Normalization is a transient process meaning there is a time window for vessel normalization to occur. For this reason, chemotherapy drugs should be administered carefully within this window to benefit from improved vascular conditions^11^. Moreover, the excessive application of antiangiogenic agents for more extended periods brings about vessel pruning, which decreases the outcome of combined therapies^12,13^. Therefore, the appropriate timing and dosing of the agents should be carefully adjusted to improve the functionality of blood vessels, as well as the delivery of anticancer drugs to tumor cells.

In cancer therapies, mathematical models are commonly studied to simulate the delivery of cytotoxic drugs and to understand the relations between tumor microenvironment and drug delivery. Different approaches to the modeling of tumor vasculature and angiogenesis have been suggested to study the applications of antiangiogenic agents combined with chemotherapy drugs. By using discrete vasculature models, the delivery of chemotherapy drugs to tumors and treatment response have been extensively investigated^14–17^. In addition, in 2D and 3D blood flow models, the use of antiangiogenic agents and their effects on tumor response has been studied by Stephanou et al.^18^. Normalization is also simulated to investigate its effects on blood flow and the combination of antiangiogenic agents with cytotoxic drugs^13,19–21^. In addition to mathematical models, angiogenesis imaging is largely studied to examine tumor growth, characterization of tumor vasculature, and the response of the therapies. Employing various imaging modalities such as computer tomography (CT) and magnetic resonance imaging (MRI) gives an insight into the progression of tumors and vascular structures^22,23^. Additionally, photoacoustic imaging is exploited to monitor tumor angiogenesis due to its higher contrast and spatial resolution^24–26^. Besides, it is feasible to detect early tumor growth and vascularization and to monitor the progress of antiangiogenic treatments^27,28^.

Early tumor response prediction, a measure of the effectiveness of treatment, is crucial in anticancer therapies to determine appropriate treatment schedules, apply the optimal drug dosages to patients, and increase patient survival. In this regard, to develop successful models for tumor response prediction, it is vital to monitor the distribution of cytotoxic drugs and to evaluate the tumor response to therapies in advance. Response prediction problems are often formulated by developing mathematical models benefiting from reaction–diffusion frameworks^29–31^. These models are capable of modeling the tumor response to an extent, but they are generally deterministic models driven by a limited number of parameters that might constrain the model to comprise the inherent tumor growth patterns. However, several studies in deep learning outperform the traditional approaches in various tasks such as tissue classification, tumor segmentation, and tumor growth prediction^32–35^. A convolutional neural network (CNN) is developed by Urban et al. to classify the images of vascular networks taken before and after various antiangiogenic drug applications in vitro^36^. In another study led by Ha et al., a CNN algorithm is used to determine the chemotherapy response prediction in patients with breast cancer. The breast MRI dataset is utilized as a baseline, and the treatment response before the application of chemotherapy drugs is predicted^37^. Positron emission tomography (PET) and CT images from different types of cancer are benefited in recent studies to develop CNN models that have the potential to predict the response of chemotherapy with high sensitivity^38,39^.

Deep learning allows us to build computational models to learn representations of data with multiple levels of abstraction for the given task^40^. For instance, a feed-forward neural network is a function approximation that maps input data to output data. The function is formed by composing simpler non-linear functions where each function provides a new representation of input data^41^. After extracting the information out of the input data, the model amplifies the essential features for a given task such as classification, segmentation, and regression. Convolutional neural networks, a neural network type, perform convolutions over the input by using the given number of filters, which can learn the spatial and temporal dynamics in the image data^40^. They are robust to overfitting due to the convolution operation properties, which reduces the full connection of the network. These models have applications in image^42^ and video recognition^43^, medical image analysis, and image processing^44^. By employing CNNs, models can be built for spatial and temporal forecasting problems in an end-to-end manner in the presence of sufficient data.

Tumor response prediction can be formulated as a spatio-temporal forecasting problem under the effects of interventions. Interventions can be described as drug injections. Therefore, the forecasting problem is equivalent to modeling the tumor microenvironment during the ongoing treatment. The tumor microenvironment can be described with tumor density, vasculature, interstitial fluid pressure (IFP), antiangiogenic treatment, chemotherapy maps, and drug dosages from the ongoing treatment. The model, denoted by *F*, takes tumor microenvironment maps ${X}_{t}$ and drug scalars ${S}_{t}$ as inputs at time *t* and predicts future tumor microenvironment maps ${X}_{t+1}$ at time ${t+1}$. The predicted future tumor microenvironment maps allow us to investigate tumor growth and shrinkage patterns as well as drug diffusion maps, which are the key indicators of treatment efficiency.

In this study, we develop a deep learning model, which aims to capture the tumor response behavior conditioned by the ongoing treatment schedule. Our study suggests that deep learning models can be helpful in assessing tumor growth and drug response in the scheduling of cytotoxic drugs. Since the required input data such as tumor density, IFP, vasculature, and drug maps is hard to collect from clinical patients for various reasons, we use the synthetic data from the mathematical model built in our previous paper^21^. Therefore, the proposed deep learning model *F* encapsulates the non-linear partial differential equations (PDEs) that govern the spatio-temporal dynamics of the tumor microenvironment. The model consists of multiple CNNs. Our motivation to use CNNs is based upon two key reasons; CNNs can extract coupled spatial features from multichannel inputs and utilize the spatial features to predict future microenvironment maps. In the end, our model uses tumor microenvironment channels and drug scalars as inputs and predicts future tumor microenvironment maps that may assist to find appropriate scheduling and dosages of the combination therapy for patients.

## Results

Our deep learning model aims to predict chemotherapy response by outputting future tumor microenvironment maps. The proposed model, Diffuser-Elapser Network (DENT), consists of seven submodels where each of which is a CNN. Model specifications and training procedures can be found in the Methods section.

The DENT model takes input tensors describing the current tumor microenvironment state, consisting of five channels, namely tumor density, vasculature, IFP, antiangiogenic drug, and chemotherapy drug maps accompanied by chemotherapy and antiangiogenic drug dosages, and predicts future tumor microenvironment maps. The model can use its predictions as its next inputs. Since the model predicts future tumor microenvironment maps, we use two image quality evaluation metrics, which are peak signal-to-noise ratio (PSNR) and structural similarity (SSIM)^45^. PSNR represents a measure of the quality of prediction by calculating the ratio between the square of maximum fluctuation among pixels in the ground truth image and MSE between ground truth and predicted images. SSIM measures the perceptual difference between two images in terms of structural information in the images. In this method, ground truth images are considered as references that have reference quality, and the quality of another image is measured by comparing it with the initial one. SSIM is mostly applied to improve or track the perceptual metrics based on the structural information; on the other hand, PSNR relies on estimating the pixel-wise error. Both metrics are unitless quantities. The PSNR score approaches infinity as the MSE approaches zero. The range for an SSIM score is the interval of $[-1,1]$. A higher score denotes better prediction quality on both metrics.

**Figure 1:**
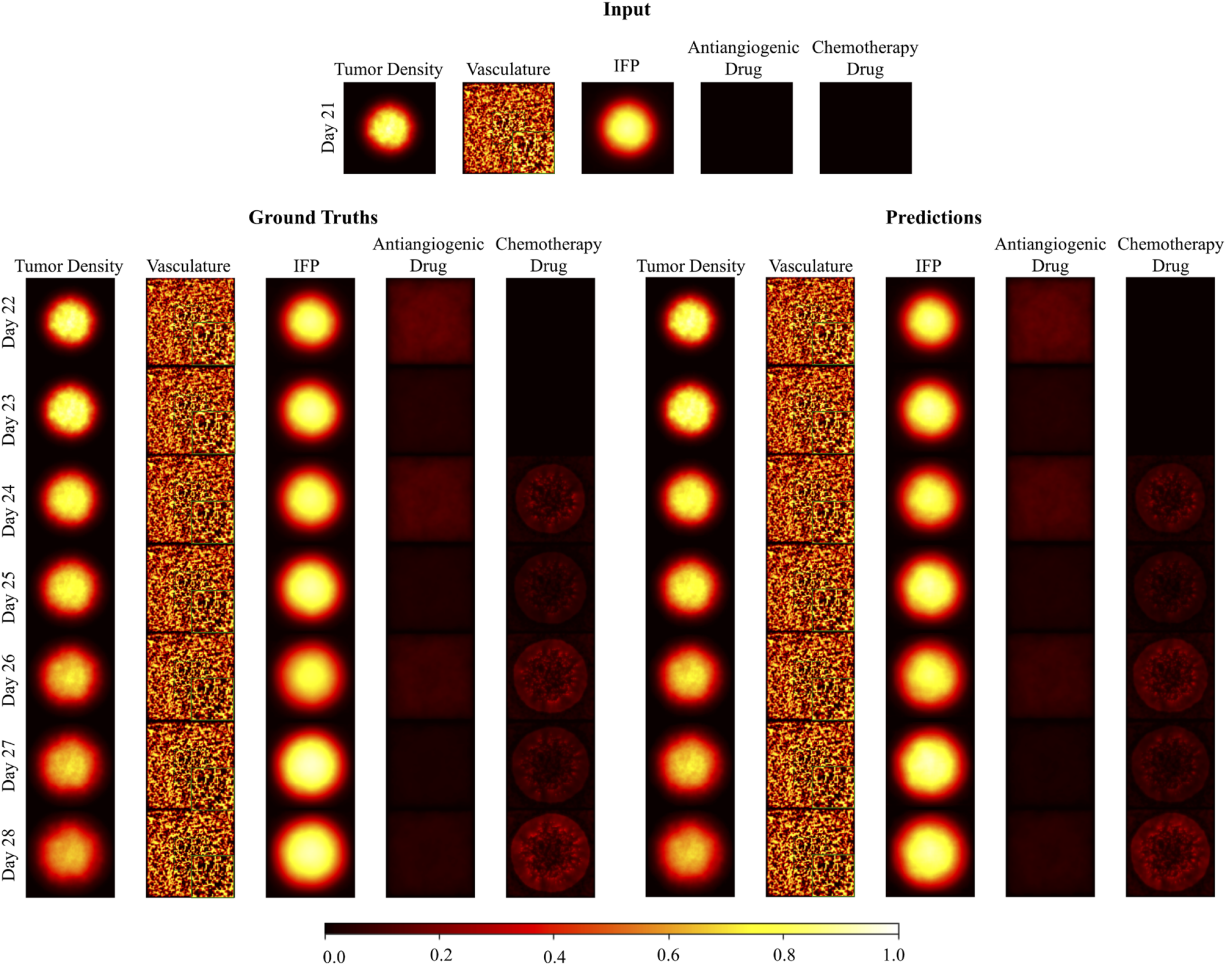
The synthetically generated tumor microenvironment maps of the example patient. The patient receives treatment between days 21 and 28. The application of the antiangiogenic drug is at the end of days 21, 23, 25, and 27 while the chemotherapy drug is applied at the end of days 23, 25, and 27. Drug scalars *A* and *d* have the values of 0.6 and 0.8 respectively. The model takes initial tumor density, vasculature, IFP, antiangiogenic drug, and chemotherapy drug maps as an input (on the top). Ground truth maps that show the ongoing treatment from day 22 to day 28 (on the left). Predicted maps that are iteratively generated from day 22 to day 28 (on the right). (In the figure, we present zoomed version of the center of the vasculature maps on the bottom right corner and the scalebar represents the scale of the pixel values of the tumor microenvironment maps.)

In the study, we first investigate the performance of the model on predicting future tumor microenvironment maps. To give an insight, we pick a synthetically generated case that contains a patient’s tumor microenvironment maps during treatment. The tumor microenvironment maps consist of five channels correspond to tumor cell density, vasculature, IFP, antiangiogenic drug diffusion, and chemotherapy drug diffusion maps. The input, ground truth, and predicted tumor microenvironment maps during the treatment are presented in Fig. 1. We prefer eight days-long cases so that we can compare our predictions with the previous simulation findings^21^, which were limited to simulating 8 days starting from the initiation of therapy. The patient receives treatment between day 21 and day 28. The application of the antiangiogenic drug is at the end of days 21, 23, 25, and 27, and the chemotherapy drug is at the end of days 23, 25, and 27. Our model takes the tumor microenvironment maps just before the initiation of therapy as input and predicts the future tumor microenvironment maps by taking its outputs as new inputs. The predicted tumor microenvironment maps show the effects of ongoing treatment on the tumor microenvironment. A qualitative assessment of Fig. 1 shows that our model successfully predicts future tumor density, vasculature, IFP, antiangiogenic drug, and chemotherapy drug maps during ongoing treatment.

In the experiments, we use 135 test cases from three different patients. The cases included five different therapy initiation days and nine different dosage combinations of antiangiogenic and chemotherapy drugs. Therefore, the cases differ according to the days of initiation of treatment, applied drug dosages and patient characteristics. A case corresponds to a tensor of 8 days-long tumor microenvironment maps accompanied by synthetically generated drug scalars using a mathematical model^21^. The consecutive frames correspond to time steps from 0 to 7 and are separated by a day. The five channels represent tumor cell density, vasculature, IFP, antiangiogenic drug diffusion, and chemotherapy drug diffusion maps. Drug scalars indicate the dosage for drug insertion in each time step if there is. The insertion schedule is the same for all cases; the antiangiogenic drug is inserted at the end of time steps 0, 2, 4, and 6, and the chemotherapy drug is inserted at the end of time steps 2, 4, and 6.

**Figure 2:**
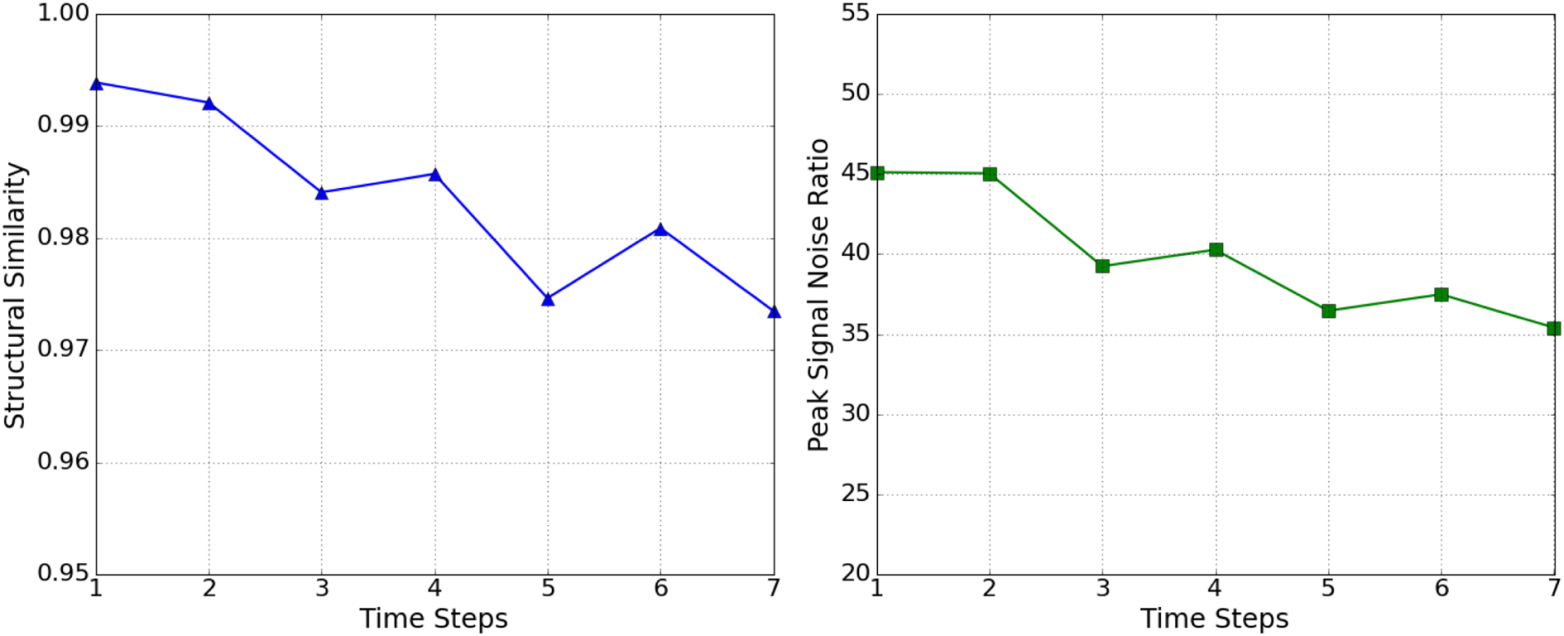
Frame wise average SSIM scores (on the left) and PSNR scores (on the right) over the 135 test cases.

By using completely new cases to test our model, we assure that the model has not see the test data before. Each forward step of the DENT model is trained to predict tumor maps in the next time step, which is a day ahead. The average PSNR and SSIM scores are shown in Fig. 2 over the test set. We see that our model manages to predict future tumor microenvironment maps up to seven days ahead with the average structural similarity score of 0.973 and the average peak signal ratio score of 35.41. The decreasing trends in SSIM and PSNR scores over time are expected as the prediction error is accumulated in each forward step in time. We also observe decreases in average SSIM and PSNR scores at time steps 3, 5, and 7 which correspond to the one-day ahead predictions following drug injections. Therefore, a possible reason behind these fluctuations may be the challenges in the drug insertion processes.

**Figure 3:**
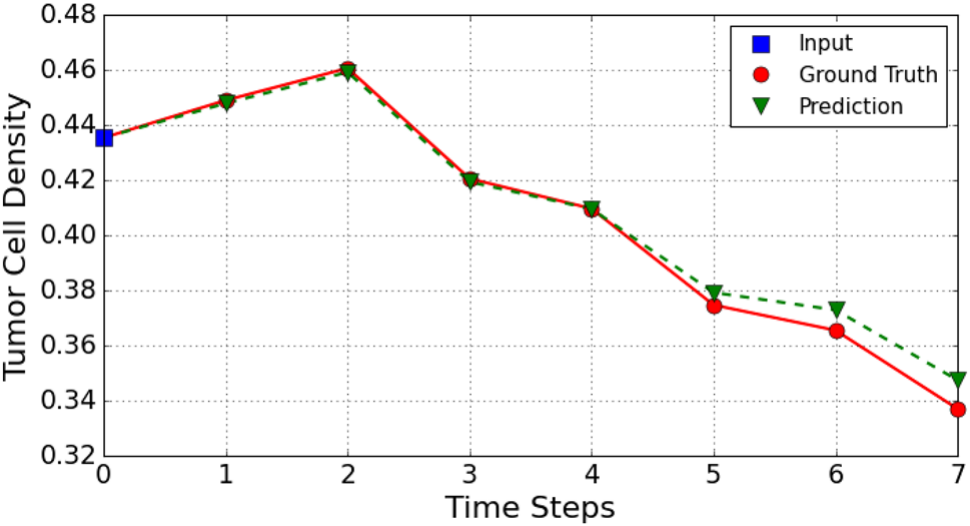
The comparison of tumor cell density values for ground truths and predictions through time steps 0 to 7 for the example case. The tumor cell density at time step 0 corresponds to the tumor cell density of input. (The application of the antiangiogenic drug is at the end of time steps 0, 2, 4, 6 and chemotherapy drug is at the end of time steps 2, 4, 6.)

We next examine if the model encapsulates the tumor growth and shrink patterns during the treatment by calculating a dimensionless metric called tumor cell density, *C*. Tumor cell density over the whole tumor shows the effectiveness of ongoing treatment. It is calculated by averaging the pixel values after thresholding the pixels of the tumor density map as performed in the previous study^21^. We compare the tumor cell density of ground truths, $C_{gt}$, and predictions, $C_{pred}$, over the time steps. For the selected patient case in Fig. 1, ground truth and predicted tumor cell density values during treatment are presented in Fig. 3. We calculate the absolute percentage error between $C_{gt}$ and $C_{pred}$ for 135 test cases which are shown in Table 1. The mean absolute percentage error at the end of day 7 is $2.292\pm 1.820$, indicating that our model encapsulates the tumor growth and shrink patterns under the effects of ongoing treatment. It can be seen that the standard deviation of error increases over time since the error accumulates in each prediction.

**Table 1:** Mean absolute percentage error (MAPE) between ground truth and predicted tumor cell densities over days for the test cases.

	Day 1	Day 2	Day 3	Day 4	Day 5	Day 6	Day 7
MAPE	$0.152\pm 0.117$	$0.198\pm 0.151$	$0.681\pm 0.584$	$1.084\pm 0.782$	$1.402\pm 1.210$	$2.048\pm 1.425$	$2.292\pm 1.820$

## Discussion

In this study, we build a deep learning model designed to solve the chemotherapy response prediction problem in a proof of concept setting. The model is trained with simulation data that reflects the biological aspects of the tumor microenvironment, including tumor growth and angiogenesis^20,21^. The parameters used in this mathematical model are calibrated and the simulation results are validated by experimental studies in the literature. In order to adjust the parameters related to the kinetics of tumor cell growth and vascularization, the experiments of Winkler et al.^8^ studying glioblastoma xenografts that grow in the mouse brain are utilized. The model has a dynamic vascular structure that shows the properties of tumor vessels and inherent vessels in the tissue. The resulting vessel density is compatible with the study of^46^, with decreasing vascular density towards the tumor center due to increased tumor cell density in that region. Moreover, it is found that IFP increases up to the MVP levels throughout the tumor and it drops sharply around the tumor rim very similar to various experimental studies (^3,4,47^). In the model, antiangiogenic agents are administered in various treatment regimens in combination with large-sized chemotherapy drugs, the distribution of which is mainly dependent on convection. Drug accumulation is mostly observed in the tumor periphery in the simulations, as there are low IFP levels in this region. The results are validated by various experimental studies examining the drug distribution of large drugs, showing that large drug accumulation is mainly observed in peripheral areas^46,48–51^. Here, the proposed deep learning model can predict chemotherapy response to tumor structure over a flexible period via iterating for a chosen number of times. Our model operates on a total of five channels: the maps of tumor cell density, vasculature, interstitial fluid pressure, and antiangiogenic and chemotherapy drug diffusions. The predicted maps show the spatial and temporal effects of ongoing treatment on tumor microenvironment maps. The model can take its outputs as inputs, go further in time, and predict the following state of the tumor by iterating this process as many times as required. In these iterations, there is an option of drug injection fed into the model as an input, if it is in the treatment schedule. To include the injection step in the model, the drug diffusion maps on the tumor structure are predicted, then these maps are used for the next tumor state prediction in combination with the other inputs such as tumor cell density, vasculature, and interstitial fluid pressure maps. To evaluate the predictive capabilities of our model, we track PSNR and SSIM scores calculated between the ground truth and predicted tumor microenvironment maps during the treatment. In a study by Teuwen et al.^52^, a deep learning model is utilized to synthesize digital breast tomosynthesis, which can be used to detect early breast cancers. When the model uses breast CT images, it has an SSIM score of 0.93. Chaudhari et al.^53^ use 3D convolutional neural networks to perform the resolution-enhancement of MRI scans. The outputs of the proposed model are compared with the other models using SSIM and PSNR metrics. A study by Zeng et al.^54^ utilizes CNNs to reconstruct super-resolution single and multi-contrast brain MRI scans. Their proposed model for multi-contrast super-resolution cases has the highest average PSNR and SSIM scores relative to the other methods. Uzunova et al.^55^ use patch-based generative adversarial networks to generate high-resolution 2D and 3D medical images. Their model achieves SSIM scores of 0.733 and 0.711 for thorax CT and X-ray datasets, respectively. Our model can predict future tumor microenvironment maps up to 7 days with the average SSIM score of 0.973 and the average PSNR score of 35.41 at the end of day 7. We have assessed the tumor cell density among the entire predicted tumor density $C_{pred}$ and compared it with the ground truth cell density $C_{gt}$. To obtain the values of cell density for both the predicted tumor and the ground truth tumor, we have used the same method as^21^, merely averaging the pixel values that are above a threshold value. We use the percentage error between $C_{pred}$ and $C_{gt}$ to evaluate the performance. The model predicts tumor cell density at the end of day 7, with the mean absolute percentage error of $2.292\pm 1.820$.

Chemotherapy response prediction aims to successfully predict whether the patient will respond to treatment, depending on the tumor characteristics of the patient. Current approaches to chemotherapy response prediction are mainly based on classification models. A recent study^56^ focuses on the prediction of lung cancer treatment response for patients treated with chemoradiation. By analyzing time-series CT images of patients, the survival probability and cancer-specific outcomes have been predicted. In another study by Ha et al.^37^, it is aimed to predict the effectiveness of neoadjuvant chemotherapy (NAC) response by using a breast MRI tumor dataset. Patients are classified into three groups as complete, partial, and no response, based on their response to NAC treatment. Although these classification-based models predict chemotherapy response to some extent, their predictions lack the spatial and temporal information that provides the necessary explanations behind the predictions. For instance, providing maps of future tumor microenvironment states after the treatment can give clinicians an insight to reschedule the treatment planning process. Thus, there is a challenge in building models that provide detailed information in the chemotherapy response prediction task, which leads us to the modeling of the tumor microenvironment.

On the other hand, mathematical models rely on nonlinear PDEs that simulate the dynamics of the tumor microenvironment while considering the effects of treatments. These models enable us to observe the diffusion of the chemotherapy drug to tumor cells via the normalized vasculature network, and the effectiveness of the applied treatment by producing maps of the future tumor microenvironment states. This approach may eliminate the main drawback of the class-based chemotherapy response prediction models, which is the lack of insight behind the predictions. A recent study^21^ presents a mathematical model to simulate the tumor state according to the previous tumor state and the drug diffusion inside the tumor. In each iteration of the model, a predetermined theoretically thirty-minute gap period proceeds and the subsequent tumor state is calculated. This allows the model to output the tumor states with a time resolution of thirty minutes, which is suitable for comprehensively studying tumor shrinkage or growth under the effects of ongoing treatment. The model is deterministic, which means differential equations, predetermined biophysical factors, and constraints are utilized. In potential clinical use, this may prevent the model from making correct interpolations since these factors and constraints may vary from patient to patient as opposed to the fixed values used in the simulation. Capturing these factors and constraints from patient data is essential to predict patient-specific chemotherapy responses. The calculations in the simulation are at the pixel level, and the model cannot capture the detailed physical structure of the tumor and recognize any special conditions, such as detecting the tumor type or distinguishing smaller parts of the tumor. Since the coefficients used in the differential equations are constant and non-learnable, a deterministic model cannot learn from clinical data and be precise enough for clinical use, making it impossible to generalize over thousands of patients.

In the wake of these limitations in the literature, we propose a deep convolutional model. The model simulates tumor microenvironment maps that encapsulate spatio-temporal effects of the ongoing treatment such as, tumor growth and shrinkage patterns, along with drug diffusion maps. The model outputs future tumor microenvironment maps that fill the descriptive information gap in classification-based response prediction models. The sequence of the predicted maps indicates the response to the therapy in the daily regimen. The model predictions reveal insights about the effectiveness of the therapy schedule and drug dosages on patients before the application of the therapy. This might allow for better adjustment of schedules, use of the potent schedules, and elimination of the ineffective ones. Although mathematical models can simulate tumor microenvironment, they tend to neglect patient-specific tumor response patterns due to a limited number of parameters, constant coefficients, and deterministic rules governing the model. Since the proposed model is built with end-to-end learning, it can learn patient-specific tumor response patterns in the presence of sufficient data. The model may improve the personalization of treatment by extracting specific clinical features of a patient and making predictions based on them.

Our model for the chemotherapy response prediction can provide insights into the treatment outcomes in clinical settings; however, some limitations should be eliminated with further study when it comes to clinical use. A limitation of the proposed model is to use 2D tumor microenvironment maps rather than 3D maps, which can better exploit spatial information of the tumor microenvironment. When the tumor microenvironment maps miss the third spatial dimension, the true nature of the tumor cannot be reflected in detail, leading to an incomplete representation of the tumor microenvironment within the model. Therefore, 3D mathematical models of tumors^57^ or 3D scanning methods such as CT are the primary candidates of data sources to solve the incomplete environment representation problem. In addition to the third spatial dimension, tumors also have a continuous structure over the time dimension, that is, a continuous course of growth and shrinkage. It may be necessary to model the time dependencies between the sequential states of the tumor, assuming that the pattern of future tumor growth or shrinkage follows its past trend under constant conditions. The input should consist of temporal information of the tumor microenvironment maps to encapsulate the effects of time dependencies on a tumor microenvironment. The model can utilize the input sequence to reveal the features of patient-specific tumor dynamics. These features can be critical to include patient-specific tumor growth patterns and tumor aggressiveness in model predictions. Although our CNN-based model captures spatio-temporal dependencies to some extent, it is preferable to use long short-term memory (LSTM)^58^ networks with sequential inputs combined with CNNs. Convolutional LSTMs^59^ can be utilized to extract both spatial and temporal dynamic changes in a single network as they are already exploited in the tumor growth prediction problem^60^. The main challenge of this approach would be to acquire regularly timed and spatially aligned scans to generate sequential data.

The simulated cases used in our study differ by therapy initiation day, drug combinations used in the therapy, and unique patients imposed by the randomly initialized vasculature maps. However, all simulated cases share the same insertion schedule in which antiangiogenic drug therapy is initiated at the beginning of the therapy, and chemotherapy is started with a two-day delay. Both drugs are inserted every other day. Since our cases lack the variations in the insertion schedules, the model may be limited in encapsulating the effects of longer or shorter time intervals between the initiation of antiangiogenic and chemotherapy treatments, or non-overlapping insertion days of the two types of drugs. A possible solution is incorporating various realistic insertion scheduling scenarios in the cases.

Obtaining the training data from simulations is beneficial in a proof of concept study; however, the use of clinical training data is inevitable for a distinguished and genuinely working model. To ensure that a deep learning model can simulate and generalize the chemotherapy response in a real scenario, it is necessary to train a model with a reasonably large amount of clinical data with sufficient variations of treatment schedules. A crucial feature of a clinically applicable response prediction model is that it can make predictions in the same domain with clinical data such as MRI, CT, and PET scans. Each scanning technique captures a unique kind of information about the tumor microenvironment, highlighting a particular aspect of the biological structure. By using these scanning techniques, it is possible to obtain the maps of tumor cells, tumor vasculature^61^, IFP^62^ and cytotoxic drug distributions^63,64^. Various scanning techniques should be combined to feed the model’s input to accurately represent the tumor, thus introducing as many aspects of the tumor as possible. This variety of scan types would provide a complete representation of the tumor within the model, boosting prediction and simulation capabilities.

## Methods

### The mathematical model for acquiring synthetic data

In this study, a mathematical model of tumor built in our previous study^21^ is used to obtain the training and testing data sets. Non-linear PDEs are written in dimensionless form and utilized in the model to mimic the tumor and its microenvironment biologically.

The model incorporates tumor cell density and vasculature as well as their interplay, and consists of tumor cell density, vasculature, IFP, antiangiogenic agent and chemotherapy drug. The reaction–diffusion equations are used to describe the spatio-temporal distribution of tumor cell density and vasculature (see Eqs. 1 and 2). Tumor cell density and vasculature are denoted by n(**x**,t) and m(**x**,t), respectively. In Eq. (1), the first term on the right-hand side defines the diffusion of tumor cell density, where $D_n$ is the diffusion coefficient, the second term models the tumor growth rate, where r is the growth rate and $n_{lim}$ is the maximum carrying capacity. When only these two terms are present, the Eq. (1) has two fixed points such that an unstable point at $n=0$ where there is not any cell population and a stable point at $n=n_{lim}$, the cell population approaches its maximal density. The third term couples the tumor cell density and vasculature in which $\alpha _{mn}$ indicates the proliferation rate of tumor cells when tumor vessels are present, and the fourth term describes the relationship between the tumor cell density and the chemotherapy drug, here $d_r$ is the rate at which tumor cells are eliminated when chemotherapy drug is applied. Initially, tumor cells are assumed to be distributed by Gaussian.

1 $$\begin{aligned} \frac{\partial n(\mathbf{x },t)}{\partial t}=D_{n}\nabla ^{2}n(\mathbf{x },t)+r_{n} n(\mathbf{x },t)(1-\frac{n(\mathbf{x },t)}{n_{lim}})+ \alpha _{mn}n(\mathbf{x },t)m(\mathbf{x },t) - d_rn(\mathbf{x },t)d(\mathbf{x },t). \end{aligned}$$

Distinct to healthy vessels, tumor vasculature is a heterogeneous structure, having vessels with tortuous and larger pores. In the model, the Eq. (2) is used to represent the heterogeneous tumor vascular network and it includes the terms for the diffusion of vessels, the production of islands of vessels, the directed motion of vasculature to tumor cells, the production of tumor vessels due to tumor-induced angiogenesis, and the elimination of vessels by application of antiangiogenic agent, respectively.

To create vessel islands, a course-grained model is utilized in the model. The term $m(\mathbf{x },t)(\alpha +\beta m(\mathbf{x },t)$ in Eq. (2) has two stable fixed points at $m=0$ and $m=1$ indicating the presence and absence of vessels, respectively. Starting from a random and positively distributed initial configuration, vessels are evolved according to Eq. (2) to randomly distributed islands. Tumor-induced angiogenesis is represented by using the term $\beta _{nm} \nabla (m.\nabla n)$ implying that the vessels move to the interior regions of the tumor.

2 $$\begin{aligned} \frac{\partial m(\mathbf{x },t)}{\partial t}= & {} D_{m}\nabla ^{2}m(\mathbf{x },t)+m(\mathbf{x },t)(\alpha +\beta m(\mathbf{x },t)+ \gamma m(\mathbf{x },t)^{2}) +\beta _{nm}\nabla .(m\nabla n)\nonumber \\&+\alpha _{nm}n(1-\frac{n(\mathbf{x },t)}{n_{lim}})m(\mathbf{x },t) - A_rm(\mathbf{x },t)A(\mathbf{x },t) . \end{aligned}$$

To describe IFP in the solid tumor, the Eq. (3) is used:

3 $$\begin{aligned} -K\nabla ^{2}P(\mathbf{x },t)=\lambda _{b}m(\mathbf{x },t) \left[ P_{v}-P(\mathbf{x },t)-\sigma _v(\pi _{c}-\pi _{i})\right] - \lambda _{\ell } P(\mathbf{x },t), \end{aligned}$$

where the term $\lambda _{b}m(\mathbf{x },t)\left[ P_{v}-P(\mathbf{x },t)- \sigma _v(\pi _{c}-\pi _{i})\right]$ is the fluid source from blood vessels to the interstitial space and $\lambda _{\ell } P(\mathbf{x },t)$ is the drainage of fluid from interstitial space to lymph vessels. In the equation, $\lambda _{b}$ and $\lambda _{\ell }$ are the hydraulic conductivities of blood and lymp vessels, respectively. The parameter $P_{v}$ is the vascular pressure, *P* is the interstitial fluid pressure, $\sigma _v$ is the osmotic reflection coefficient. The terms $\pi _{c}$ and $\pi _{i}$ indicate the capillary and the interstitial oncotic pressures.

The transport of the antiangiogenic agents is represented by the following diffusion equation (4). Here, the first term on the right-hand side is the diffusion of the antiangiogenic agents, where $D_A$ is the diffusion coefficient of the agents in tissue. The second term is the diffusion of the agents through vessels where $\lambda _A$ is the transvascular diffusion coefficient of antiangiogenic agents, and $A_v$ is the concentration of the agents in plasma. The third term is the drainage of the agents into the lymphatics, and the last term is the decay of antiangiogenic agents in tissue, where $k_A$ is the natural decay rate.

4 $$\begin{aligned} \frac{\partial A(\mathbf{x },t)}{\partial t}=D_{A}\nabla ^{2}A(\mathbf{x },t)+ \lambda _{A}m(\mathbf{x },t)(A_{v}-A(\mathbf{x },t))- \Gamma _{l}A(\mathbf{x },t)-k_{A}A(\mathbf{x },t) . \end{aligned}$$

A convection–diffusion equation is used to present the delivery of chemotherapy drug molecules, which are larger particles (around 100 nm in size). In the Eq. (5), the first and second terms define the diffusion and convection of chemotherapy drugs in the tissue where $D_d$ is the diffusion coefficient of drugs, and $k_E$ is the retardation coefficient for convection in the interstitium. The third term is the convection of drugs through the vessels, where $\sigma _{d}$ is the solvent drag reflection coefficient, the fourth and fifth terms are the drainage of chemotherapy drugs to the lymph vessels and the reaction of chemotherapy drugs with tumor cells, where $d_r$ is the reaction rate. The last term represents the decay of drugs in tissue, where $k_d$ is the natural decay rate.

5 $$\begin{aligned} \frac{\partial d(\mathbf{x },t)}{\partial t}=D_{d}\nabla ^{2}d(\mathbf{x },t)+{\nabla }\cdot \left( k_Ed(\mathbf{x },t) K{\nabla }P\right. ) +\Gamma _{b}(1-\sigma _{d})d_{v}- \Gamma _{l}d(\mathbf{x },t) -d_{r}d(\mathbf{x },t)n(\mathbf{x },t)-k_{d}d(\mathbf{x },t), \end{aligned}$$

Equations (4) and (5) are solved in steady-state since the time scale for the transport of antiangiogenic agent and chemotherapy drug is shorter than the time scale of the tumor growth. Antiangiogenic agent and chemotherapy drug are applied with bolus injection through an exponential decay function:

6 $$\begin{aligned} A_{v}\!\left( t\right) =A_{0}e^{-t/t^{A}_{1/2}}, \end{aligned}$$

7 $$\begin{aligned} d_{v}\!\left( t\right) =d_{0}e^{-t/t^{d}_{1/2}}, \end{aligned}$$

where $A_0$, $d_0$, $t^A_{1/2}$ and $t^d_{1/2}$ stand for the initial plasma concentration and the plasma half-life of antiangiogenic agent and chemotherapy drug, respectively. No-flux boundary conditions are applied for antiangiogenic agent and chemotherapy drug.

### Datasets

The datasets used in this study are synthetically generated with a MATLAB simulation^21^ which mathematically models the tumor microenvironment, as described in the previous section. The simulation results are consistent with experimental studies, allowing them to be used for data generation^46,48,50,51,65^. Two datasets are used in the training of the proposed deep learning model DENT. In the training and validation sets, cases from five unique patients are used. A case corresponds to tumor microenvironment maps during eight days of combination therapy. The cases contain tumor microenvironment maps with a time resolution of 30 minutes and have five different therapy initiation days in the range of day 14 to day 21. The combinations of three different antiangiogenic and chemotherapy drug dosages are used in treatments. The insertion schedule is the same in all treatments. The antiangiogenic drug is inserted at the end of time steps $t_{0}$, $t_{2}$, $t_{4}$, $t_{6}$ and chemotherapy drug is inserted at the end of time steps $t_{2}$, $t_{4}$, $t_{6}$ where $t_{0}$ denotes the starting day of therapy. Considering the initiation days and drug dosage combinations, each patient has 45 cases. Training and validation datasets are formed by utilizing 225 synthetically generated cases with five unique patients.

**Figure 4:**
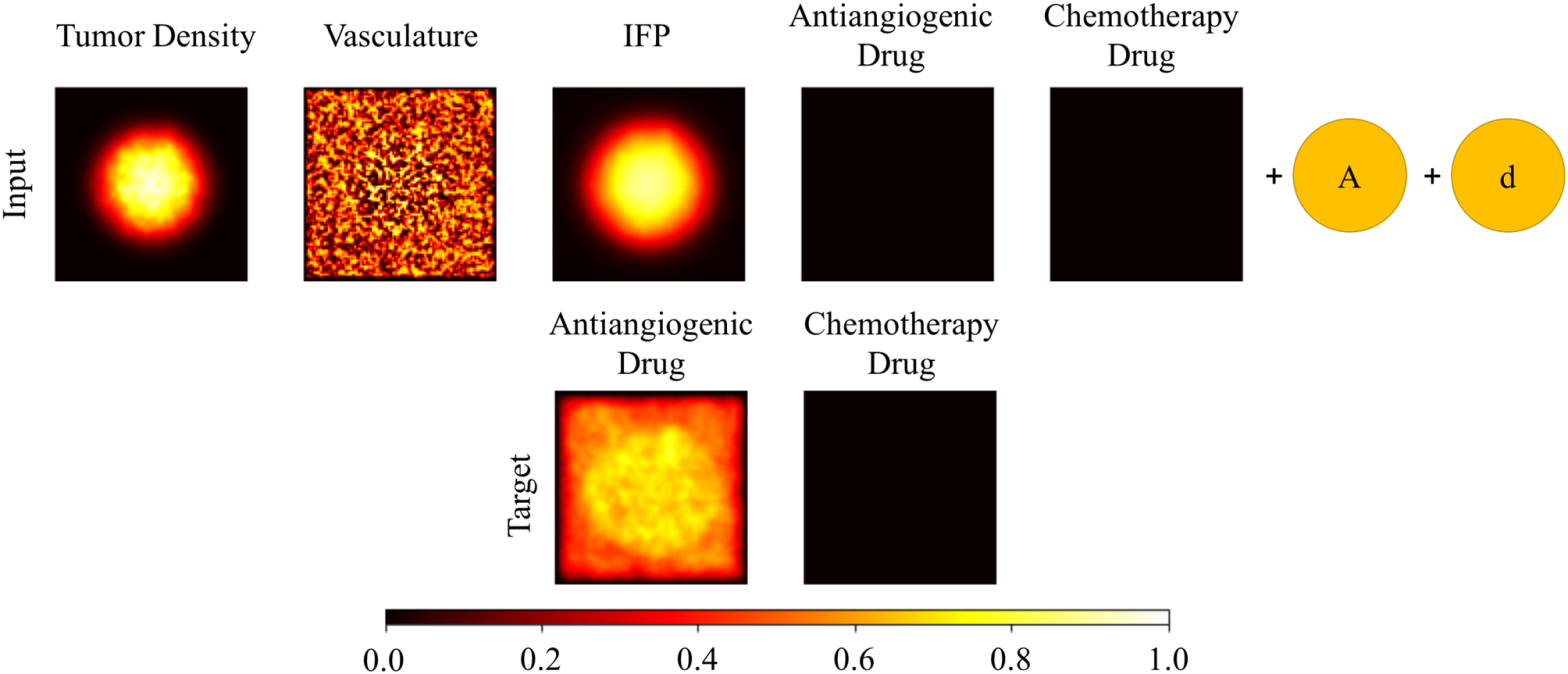
An example pair from the Diffuser dataset is shown. The input and target tensors are presented in first and second rows respectively. The input tensor consists of five channels which are tumor density, vasculature, IFP, antiangiogenic drug, and chemotherapy drug maps accompanied by antiangiogenic drug dosage (A) and chemotherapy drug dosage (d). The target tensor consists of two channels, the antiangiogenic drug and chemotherapy drug maps. The time interval between input and target tensor is one hour.

The first dataset, employed by Diffuser, contains drug map changes in the presence of drug insertion, a combination of antiangiogenic and chemotherapy drugs (Fig. 4). This dataset consists of 900 pairs. The first element is the tumor microenvironment tensors, accompanied by drug scalars that represent the dosage of the administered drug. The second element is the drug diffusion maps tensor immediately after drugs are diffused. The size of tumor microenvironment maps in pairs is $151\times 151$. It should be noted that we only consider the changes in drug diffusion maps between input and target tensors. Therefore, the time interval between the input and output tensors corresponds to an hour in which drugs are fully diffused in the tumor microenvironment. Using these pairs in the Diffuser dataset, we train our model to approximate another mapping function between input and target tensors, which is equivalent to the learning dynamics of drug diffusion given the tumor microenvironment maps and drug dosage scalars. Since there is not any considerable change in tumor density, vasculature, and IFP maps within an hour, we omit these channels in the target tensor.

**Figure 5:**
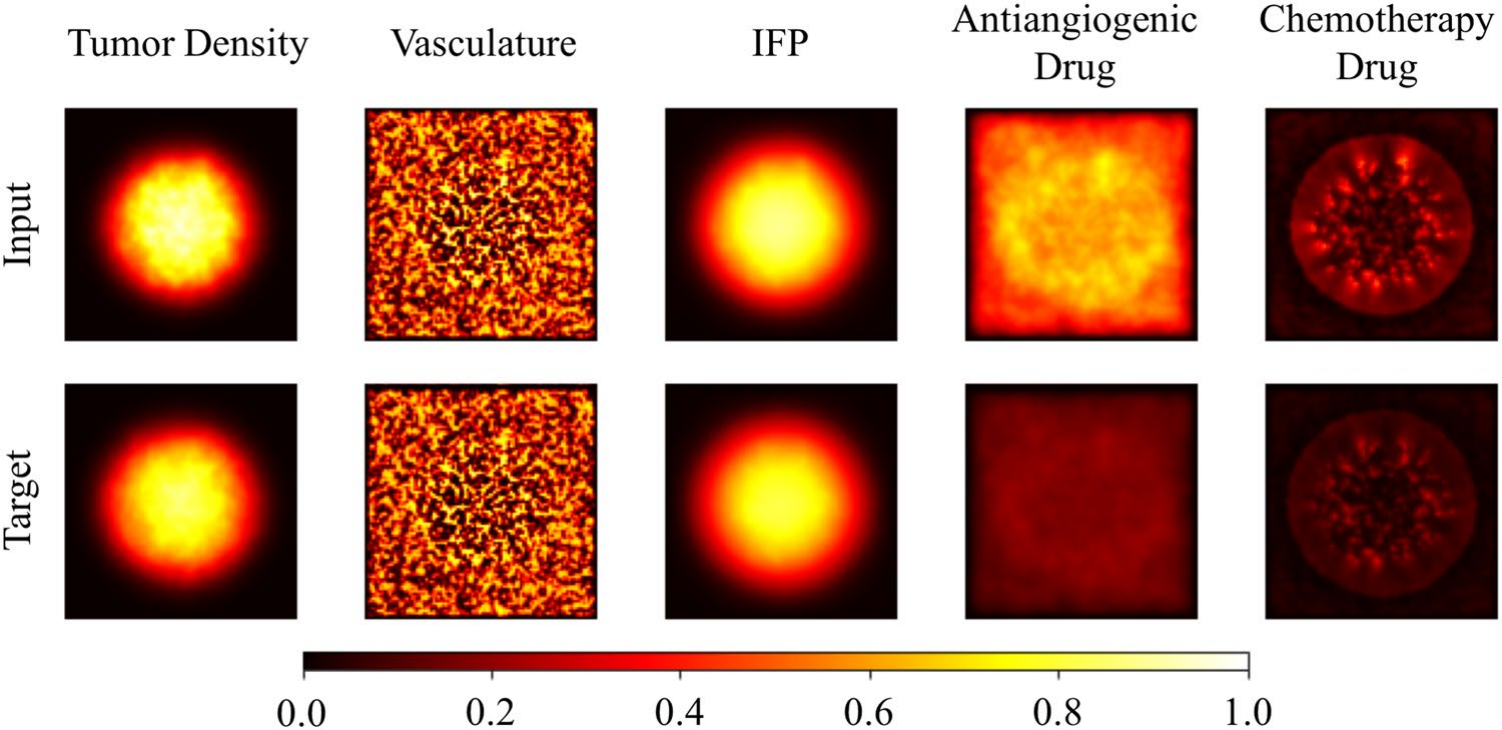
An example pair from the Elapser dataset is shown. The input and target tensors are presented in the first and second rows, respectively. Each tensor consists of five channels: tumor density, vasculature, IFP, antiangiogenic drug, and chemotherapy drug maps. The time interval between the input and the target tensor corresponds to one day.

The second dataset, used by Elapser, contains the changes in the tumor microenvironment in the absence of drug insertion between the input and target tensors (Fig. 5). The dataset consists of 1575 well-separated pairs of tumor microenvironment maps. The first element of the pair is the input tensor, and the second element is the target tensor. Both tensors comprise five channels sized by $151\times 151$, which are tumor density, vasculature, IFP, antiangiogenic drug, and chemotherapy drug maps. Elements in a pair are separated by a day apart. Using these pairs, we train our model to approximate a mapping function that maps the input tensors to the target tensors. Since the time interval between the input and target tensors corresponds to a day, the task is equivalent to learning the dynamics of tumor states in a daily regimen.

### Data preprocessing

We aim to eliminate any numerical instabilities between the pixel values of the input channels and the drug scalars. All input images and scalars in the dataset are scaled with min–max scaling using the following function:

8 $$\begin{aligned} z_{i}=\frac{x_{i}-\min (x)}{\max (x)-\min (x)} \end{aligned}$$

In the case of image inputs such as tumor density, vasculature, IFP, antiangiogenic and chemotherapy drug maps, $x_{i}$ is the pixel value, $\max (x)$ is the maximum pixel value, and $\min (x)$ is the minimum pixel value in the interesting channel. In the case of scalar inputs such as antiangiogenic and chemotherapy drug dosages, $x_{i}$ is drug dosage value, $\max (x)$ is the maximum value, and $\min (x)$ is the minimum value from the corresponding drug. The normalized value of $x_{i}$ corresponds to $z_{i}$. After scaling, each pixel and scalar input is within the range of [0, 1]. It should be noted that each $\max (x)$ and $\min (x)$ value is derived from the training set to ensure that we do not use any information obtained from the test set.

### The deep learning model

#### Functionality of simulating for flexible period of time

The proposed model takes the tumor microenvironment maps $X_{t}$ and drug scalars $S_{t}$ and predicts the future microenvironment maps $X_{t+1}$ at each forward pass. The time difference between *t* and $t+1$ corresponds to one day. Since $X_{t}$ and $X_{t+1}$ are both tumor microenvironment maps, we can feed the model with its prediction $X_{t+1}$ and $S_{t+1}$ to generate $X_{t+2}$. This process can flexibly go on to obtain the tumor state at any time point $X_{t+x}$, where *x* is arbitrary and indicates the number of prediction steps.

#### DENT model as the composition of Diffuser and Elapser models

The DENT model has the task of predicting future tumor microenvironment maps considering the effects of ongoing treatment. The model consists of multiple CNN submodels to extract spatial features from multichannel tumor microenvironment inputs. The model utilizes these features to predict future tumor microenvironment maps, which is equivalent to learning the spatio-temporal dynamics of the tumor microenvironment. The model has two main tasks; the first task is to insert and diffuse the administered drug dosages in the given tumor microenvironment, and the second task is to predict future tumor microenvironment maps. Since these tasks are not overlapping, we build two submodels called Diffuser and Elapser networks that work independently of each other. These two models are integrated to obtain the DENT network which can iterate by taking its outputs as inputs. It can check if there is any injection and interfere with the iteration process in case of necessity.

The first part of the proposed model, the Diffuser network, contains two independent CNN submodels that insert and diffuse the scalar drug dosages given together with the input tumor microenvironment maps, generating new drug diffusion maps. It is a conditional network that is initiated if scalars are different from zero. In the case of zero drug dosage, the Diffuser network outputs the relevant drug map without any changes. The second part, the Elapser model, includes five independent CNN submodels that take tumor microenvironment maps as input tensor and predict future tumor microenvironment maps in a channel-wise manner. The Elapser model encapsulates the changes in each channel so that it can extrapolate to the future.

**Figure 6:**
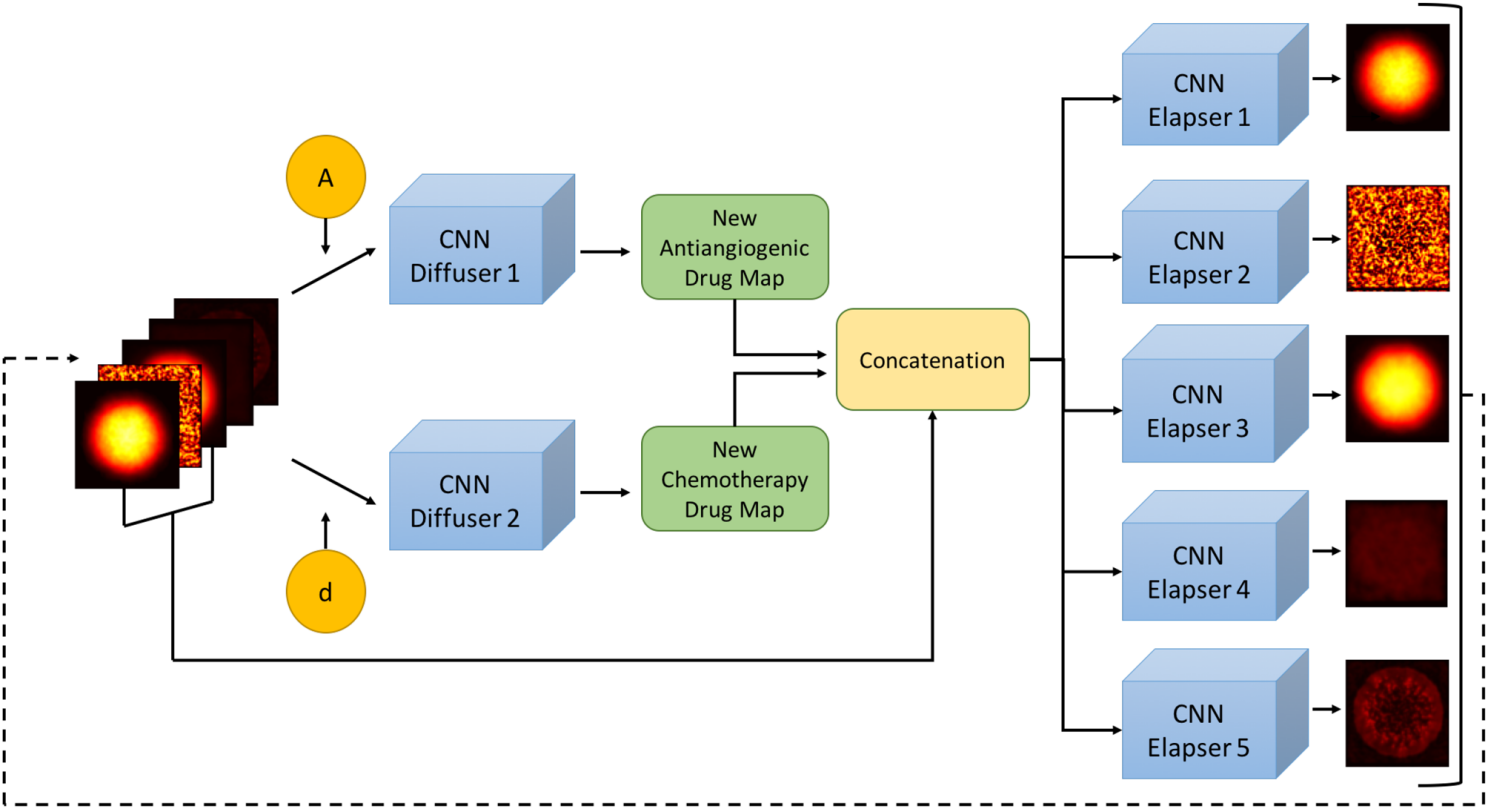
The model diagram. The model takes the five-channel tumor microenvironment maps with antiangiogenic drug dosage (A) and chemotherapy drug dosage (d). The Diffuser submodel predicts new antiangiogenic and chemotherapy drug diffusion maps, then tumor density, vasculature, and IFP maps are concatenated with new drug diffusion maps. The Elapser submodel takes the concatenated tumor microenvironment maps and predicts tumor microenvironment maps of the next day. The model can predict tumor microenvironment maps over time, as it has the capacity to use the final prediction as an input.

By creating a pipeline with the Diffuser and Elapser networks as shown in Fig. 6, we build a deep learning model that iterates for a given number of time steps. It takes its outputs as the inputs of the next step while iterating, which is suitable for performing intervention due to the drug insertion schedule.

### Model specifications and training procedures

As previously mentioned, the proposed pipeline consists of two submodels. The first submodel, Diffuser network, injects and diffuses the scalar drug dosage given the input tensor, generating new antiangiogenic and chemotherapy drug diffusions maps. An input tensor for the Diffuser network contains tumor microenvironment maps accompanied by two dosage scalars, as shown in Fig. 4. We first tile these scalars to our input tensor so that the input has the shape of $7\times 151\times 151$. The Diffuser network includes two CNNs that are trained separately with the same input tensors. The first CNN approximates a new antiangiogenic drug diffusion map, and the second CNN approximates a new chemotherapy drug diffusion map. The second submodel, Elapser network, forecasts the future tumor microenvironment maps by extrapolating from the input tensor shaped $5\times 151\times 151$ shown in Fig. 5. It outputs future tumor microenvironment maps as a five-channel image tensor. There is a separate CNN block to predict each channel of the future image tensor.

All CNN submodels are trained separately as their tasks differ from each other. In the training, we use 225 synthetically generated cases from five unique patients. We perform a 5-fold cross-validation among the five patients. Therefore, there is not any spatial or temporal dependency between the training and validation sets. The submodels are trained with MSE loss for 200 epochs with early-stopping to avoid over-fitting. The activation function is ReLu and kernel size fixed as $5\times 5$, whereas stride length is 1. After each layer, 2D batch normalization is performed except the final layer. We use ADAM^66^ optimizer with a batch size of 10 and initial learning rate of $10^{-4}$. The number of layers and filters is selected based on the best performance, and increasing the depth of the network or the number of filters does not affect model performance. The model specifications are shown in Table 2. All models are built with Pytorch^67^ backend and trained on a single NVIDIA Tesla K80 GPU.

**Table 2:** Model specifications.

Submodel(s)	Hidden channels
Diffuser 1–2	128, 64, 32, 16, 8, 1
Elapser 1–2–3	128, 64, 32, 16, 8, 4, 1
Elapser 4–5	256, 128, 64, 32, 16, 8, 4, 1

## Supplementary Information

Supplementary Information.
