# Supplementary material for: Chemotherapy response prediction with diffuser elapser network
Source: Sci Rep. 2022 Jan 31;12:1628. doi: 10.1038/s41598-022-05460-z (PMC8803972; doi:10.1038/s41598-022-05460-z)
Supplement: Supplementary file 1 — Supplementary Information. [file 41598_2022_5460_MOESM1_ESM.pdf]

Chemotherapy Response Prediction with Diffuser Elapser Network

Batuhan Koyuncu[1,4,+], **Ahmet Melek**[2,4,+], **Defne Yilmaz**[3,4,+], **Mert Tuzer**[3,4,+], **and Mehmet Burcin Unlu**[3,4,5,*]

[1]Bogazici University, Department of Computer Engineering, Istanbul, 34342, Turkey
[2]Bogazici University, Department of Management, Istanbul, 34342, Turkey
[3]Bogazici University, Department of Physics, Istanbul, 34342, Turkey
[4]Bogazici University, Center for Life Sciences and Technologies, Istanbul, 34342, Turkey
[5]Hokkaido University, Global Station for Quantum Medical Science and Engineering, Global Institution for Collaborative Research and Education (GI-CoRE), Sapporo, 060-8648, Japan
[*]burcin.unlu@boun.edu.tr
[+]these authors contributed equally to this work

Supplementary Information

We demonstrate how the proposed model can be used for deciding appropriate regimens by forwarding our model with different drug dosages. For the sake of completeness, we demonstrate our results in Figure S1 which shares the input state with the example case shown in Figure 1 of the main manuscript. In Figure S2, we present tumor cell density values that correspond to the experiments in Figure S1. These results provide qualitative and quantitative metrics for deciding appropriate drug dosages.

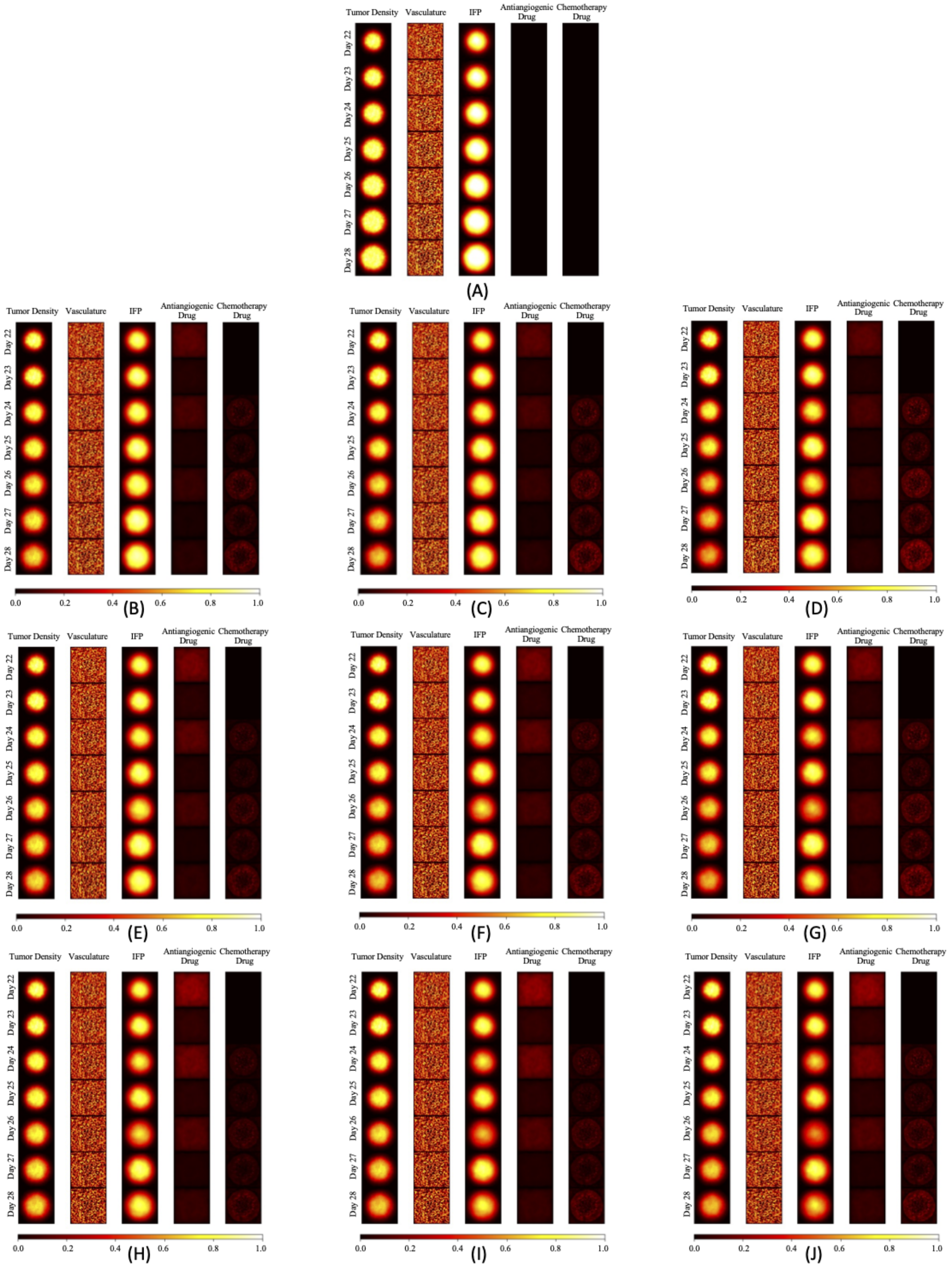

Figure S1. Ten model experiments with different drug dosages. The network input state and treatment schedule are the same as the one in Figure 1 of the main manuscript. Experiment (A) has no drugs, (B) has $A = 0.6$ $d = 0.6$, (C) has $A = 0.6$ $d = 0.8$, (D) has $A = 0.6$ $d = 1$, (E) has $A = 0.8$ $d = 0.6$, (F) has $A = 0.8$ $d = 0.6$, (G) has $A = 0.8$ $d = 1$, (H) has $A = 1$ $d = 0.6$, (I) has $A = 1$ $d = 0.8$, (J) has $A = 1$ $d = 1$.

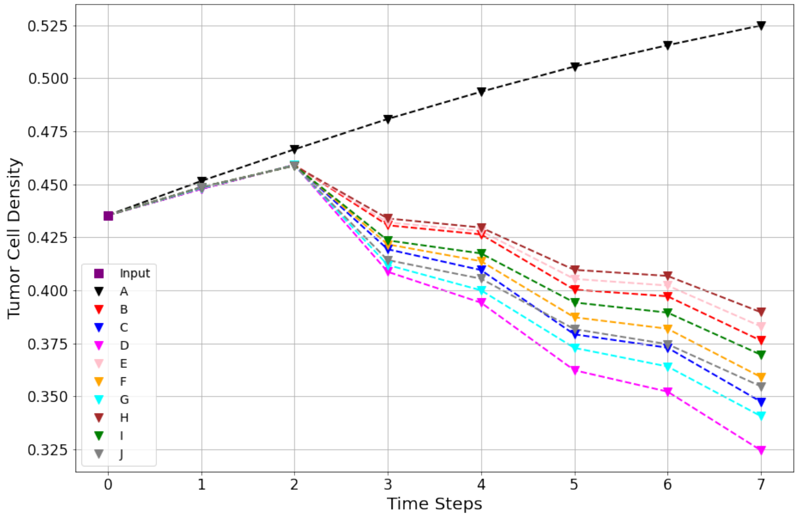

Figure S2. Tumor cell density for ten experiments in Figure S1 over 8-days long treatment.
